# The medicinal mushroom *Ganoderma lucidum* prevents lung tumorigenesis induced by tobacco smoke carcinogens

**DOI:** 10.3389/fphar.2023.1244150

**Published:** 2023-09-06

**Authors:** Ayaz Shahid, Mengbing Chen, Steven Yeung, Cyrus Parsa, Robert Orlando, Ying Huang

**Affiliations:** ^1^ Department of Pharmaceutical Sciences, College of Pharmacy, Western University of Health Sciences, Pomona, CA, United States; ^2^ College of Osteopathic Medicine of the Pacific, Western University of Health Sciences, Pomona, CA, United States; ^3^ Department of Pathology, Beverly Hospital, Montebello, CA, United States

**Keywords:** lung cancer, *Ganoderma lucidum*, benzo[a]pyrene, 4-(methylnitrosamino)-1-(3-pyridyl)-1-butanone, chemoprevention

## Abstract

*Ganoderma lucidum* (GL)*,* commonly known as “Lingzhi”, is a well-known medicinal mushroom with antioxidant and anti-cancer activity. This study examined the effects of a commercial GL product (GLSF) containing the spore and fruiting body in a 30:8 ratio on tobacco smoke carcinogen-induced lung toxicity and carcinogenesis. The potential chemopreventive effect of GLSF was evaluated *in vitro* and *in vivo*. The non-tumorous human bronchial epithelial cells (BEAS-2B cells) were treated with GLSF extract (0.025 and 0.05 mg/mL), which significantly blocked malignant transformation induced by benzo[a]pyrene diol epoxide (BPDE) in a dose-dependent manner. To confirm its anti-carcinogenic activity *in vivo*, the mice were pre-treated with GLSF (2.0 g/kg of body weight) or curcumin (100 mg/kg of body weight) by oral gavage daily for 7 days and then exposed to a single dose of benzo[a]pyrene (B[a]P) (125 mg/kg of body weight). The GLSF-treated mice showed a significant reduction in B[a]P-induced lung toxicity, as indicated by decreased lactate dehydrogenase activity, malondialdehyde levels, inflammatory cell infiltration, and improved lung histopathology. We next determined the chemopreventive activity of GLSF in mice which were exposed to two weekly doses of 4-(methylnitrosamino)-1-(3-pyridyl)-1-butanone (NNK, 100 mg/kg, on the 1st and 8th days) and fed with control or a modified diet containing GLSF (2.0 g/kg) or metformin (250 mg/kg) for 33 weeks. The GLSF and metformin treatments blocked NNK-induced lung tumor development by decreasing the lung weight, tumor area, and tumor burden compared to the mice exposed to NNK only. GLSF treatment also attenuated the expression of inflammatory, angiogenic, and apoptotic markers in lung tumors. Therefore, GLSF may be used for ameliorating tobacco smoke carcinogens-induced lung toxicity and carcinogenesis.

## Introduction

Cancer is among the leading cause of death worldwide, and in 2022, around 609,360 people died of cancer in the US (Siegel et al., 2022). Approximately 350 people die daily because of lung cancer, nearly 2.5 times more than colorectal cancers (Siegel et al., 2023). In 2023, about 103,000 lung cancer deaths will be caused by tobacco smoking which is around 81% of total lung cancer death (Siegel et al., 2023), and 3,560 deaths were caused by second-hand smoking (Islami et al., 2018). Since tobacco smoking is one of the most common causes of lung cancer death, developing a preventive strategy against tobacco-mediated lung carcinogenesis is necessary. The tobacco components 4-(methylnitrosamino)-1-(3-pyridyl)-1-butanone (NNK) and benzo(a)pyrene (B[a]P) have been widely investigated for their carcinogenic nature and their role in lung cancer development. Both carcinogens highlight the significance of understanding the harmful components of tobacco smoke and the need for preventive measures to reduce exposure to these carcinogens (Pfeifer et al., 2002; Narayanapillai et al., 2014). The NNK and B[a]P are procarcinogens which are transformed into reactive metabolites by phase 1 and phase 2 enzymes and then cause DNA mutations that can lead to lung cancer (Moorthy et al., 2015). The National Institutes of Health (NIH) estimates that the cancer care costs in the US were about $147.5 billion in 2015, among which $13.4 billion was mainly due to lung cancer (Walter et al., 2018). Conventional cancer therapies are limited by drug resistance and adverse effects, and therefore, natural products obtain attention as candidates for lung cancer prevention. Therefore, developing effective and safe agents to inhibit tobacco smoke carcinogen-mediated lung carcinogenesis as preventive medicines are highly urgent.

Around 3,000 plant species have been shown to contain active components with anti-cancer activity, and about 30 compounds derived from natural products have already been examined in clinical studies against different diseases (Gali-Muhtasib et al., 2015; Atanasov et al., 2021). Ganoderma lucidum (Curtis) Pleurotus karst (GL) a medicinal mushroom, has been used for decades as a nutraceutical to improve health and treat numerous chronic and infectious diseases (Bishop et al., 2015). In addition to being an anti-cancer agent, studies have also shown that GL can act as an immunomodulator (Atanasov et al., 2021; Shahid et al., 2022). GL is also a type of traditional Chinese medicine (TCM) that can be consumed in various forms, including powders, decoctions, and extracts (Liang et al., 2019). It is often combined with other herbs to create synergistic effects that address specific health diseases. Typically, GL is used in TCM herbal formulas, such as FEI YANNING Decoction, which includes GL alongside other medicinal herbs (Wang et al., 2009; Guo et al., 2012; Hoffman et al., 2020). Studies have shown that this formula could potentially benefit cancer treatment (Wu et al., 2022). It is important to note that besides FEI YANNING, there are many other classic TCM formulas that contain GL, such as the FUZHENG YILIU Decoction (Chen et al., 2014). The polysaccharides and triterpenoids that are isolated from the fruiting body, mycelium, or spores of GL, are investigated for treating different diseases, including cancer (Zhu et al., 2013; Moorthy et al., 2015). In addition to anti-cancer activities, GL can modulate oncogenic signaling pathways, such as the PI3K/Akt/mTOR and JAK/STAT5 in cancer (Weng and Yen, 2010; Gill and NavgeetKumar, 2017; Jin et al., 2020). However, the effects of GL on tobacco smoke carcinogens-mediated toxic and carcinogenic effects in the lung have not been thoroughly investigated.

The present study aimed to determine whether a specific GL product GLSF can prevent tobacco smoke carcinogen-induced lung carcinogenesis. First, we examined the effects of GLSF on the malignant transformation of the non-tumorous human bronchial epithelial cells *in vitro*. Next, we examined the protective activity of GLSF against single dose B[a]P-induced short-term biomarkers of lung toxicity in mice. Eventually, we demonstrated the chemopreventive efficacy of GLSF in a mouse model of lung carcinogenesis induced by NNK. These data may provide pre-clinical evidence that the GLSF can be used as preventive therapy for individuals with an increased risk of lung cancer, such as heavy smokers. Since GLSF is a dietary supplement without major side effects, the outcome of this work should be readily translated into a clinical lung cancer chemoprevention regimen.

## Materials and methods

### Compounds and reagents

This study used a specific commercial product manufactured by Beijing Tong Ren Tang Chinese Medicine Co. (Hong Kong, China)., named GLSF, which contains a mixture of the spore and fruiting body of GL, to evaluate a commercially available product which is under strict quality control. GLSF in powder formulation was provided by Beijing Tong Ren Tang Chinese Medicine Co. The chemical component analysis for GLSF has been reported before (Liu et al., 2021).

## Preparation of GLSF extracts

Based on the protocol developed by (Liu et al., 2021) the GLSF extract was prepared. After using the first 5 g of spore and fruiting body of GL, a lyophilized powder weighing 2.772 g was produced. This resulted in a yield of 55.44%. This GLSF powder was mixed with 50 mL of artificial gastric juice and incubated for an hour at 37°C while shaking. Then, 50 mL of artificial intestinal juice was added, and the mixture was incubated for another 5 hours with shaking at 37°C. After centrifugation at room temperature for 15 min at 4,500 g, the supernatant was collected. The extract was neutralized to pH 7.0 with 0.2 M NaOH, filtered using Whatman filter paper, lyophilized, and stored at −20°C. The major active components were determined using a validated HPLC-DAD method (Yeung et al., 2022), which was able to quantify 13 major components in GLSF including ganoderenic Acid C, ganoderic Acid C2, ganoderic Acid G, ganoderic Acid B, ganoderenic Acid B, ganoderic Acid A, ganoderic Acid H, ganoderenic Acid D, ganoderic Acid D, ganoderic Acid F, ganoderic Acid DM, ganoderol A, and ergosterol. The individual contents in the extracts were quantified and used as a fingerprint for GL products.

### Cell culture

The BEAS-2B cells, which are human bronchial epithelial cell lines that have been immortalized by SV40, were purchased from the American Type Culture Collection (ATCC) located in Manassas, VA. The ATCC website provided instructions for culturing the cells. To maintain the cells, used the Airway Epithelial Cell kit with basal medium and supplements from PromCell, and placed in fibronectin-coated flasks at 37°C. For subculturing, using a solution of 0.25% Trypsin and 0.53 mM EDTA, which also contained 0.5% polyvinylpyrrolidone (PVP) from Sigma.

### SRB assay

For SRB assay, we seeded 3,000 cells in 96 plates wells plate. After overnight incubation, we treated them with drugs for 72 h at 37°C in a 5% CO_2_/95% air. Next, we fixed the cells overnight using 10% trichloroacetic acid and stained them with SRB Sodium Salt from Sigma-Aldrich. We removed excess staining solution with 1% acetic acid and allowed the fixed cells to dry. Finally, we dissolved the dye using a 10% tris base solution and used a µQuant Microplate Reader from BioTek Instruments in Santa Clara, CA, to read the results.

### Cell transformation and colony formation assay

To determine cellular transformation, the soft agar assay protocol was used with some modifications as described previously. (16). In brief, BEAS-2B cells at a density of 500 cells per cm^2^ in 75 cm^2^ dishes has been seeded with complete media. After 24 h, treated the cells with a single dose of BPDE (0.2 μM) for 1 h, washed them with DPBS, and replaced it with complete media. Then cultured the cells for 5–7 days until they reached about 80% confluence. Next, plated the cells for soft agar assay, mixing 2000 cells per well with 0.33% noble agar (Sigma) in complete media in a 96-well tissue culture plate, layered over a solidified bottom layer containing 0.5% agar in complete media. To evaluate the effects of carvedilol, we pre-treated the cells with various drug concentrations for 2 h before exposing them to BPDE. After BPDE exposure, added fresh media containing GLSF to the culture and also added it to the top layer of the agar mixed with the cells. Then incubated the plates at 37°C with 5% CO2/95% air for 7–10 days and counted the colonies under a microscope that was larger than ten cells.

### Rodent diet preparation

To conduct *in vivo* studies, GLSF powder was added to mouse diets for oral consumption in a concentration of 1.25%. The modified animal diet was obtained from Newco Distributors, Inc. (Rancho Cucamonga, CA) and comprised of 98.45% Laboratory Rodent Diet #5001, 1.25% GLSF, and 0.3% color dye. The dosage calculation was based on an average daily intake of 4 g per mouse, with a targeted 2 g/kg dose. The animal diets were stored at 4°C upon delivery, and food consumption for each group was monitored throughout the study. The results confirmed that the GLSF dose administered was approximately 2.0 g/kg.

### Animal studies

The Institutional Animal Care and Use Committee (IACUC) at Western University of Health Sciences approved all animal studies conducted. The mice used in this study had unrestricted access to water and food and were kept in a temperature-controlled facility with 35% humidity, following a 12-h light and dark cycle.

### Acute short-term B[a]P exposure mouse study

The treatment regimen (Figure 2A) of the prophylactic effect of GSLF against lung toxicity was selected based on studies in our lab, while the dose of B[a]P was chosen in accordance with a previous study (Sehgal et al., 2013). Twenty male CD1/IGS mice were randomly allocated into four groups having five animals in each group. Group I: Served as control and administered vehicle by oral gavage for 7 days and corn oil orally on the 7th day only. Group II: Served as a toxicant group and received only a single dose of B[a]P (125 mg/kg in corn oil) by oral gavage on the 7th day. Group III and Group IV: Pre-treated with curcumin (100 mg/kg) and GSLF (2 g/kg) by oral gavage daily from day 1 to day 7, and B[a]P (125 mg/kg in corn oil) was given on the 7th day orally 2 h after the pretreatment with curcumin and GSLF. Animals were euthanized 24 hours after the B[a]P treatment. Blood samples were collected via cardiac puncture. The lungs were perfused with saline before being harvested.

#### Lactate dehydrogenase (LDH) activity assay

To confirm lung damage caused by B[a]P, conducted an LDH assay using a lactate dehydrogenase activity assay kit (MAK066, Sigma-Aldrich). Following the manufacturer’s protocol, we used 25 µL of mouse plasma, and the resulting LDH concentration was expressed as miliunits/mL, in accordance with established methods (Dutta et al., 2018).

#### Lipid Hydroperoxide (LPO) assay

To determine lipid peroxidation, used a thiobarbituric acid reactive substances (TBARS) assay from Cayman Chemical in Ann Arbor, MI, United States (product no. 10009055). Following the manufacturer’s protocol and expressed the concentration of thiobarbituric acid-reactive species as malondialdehyde (MDA) in µM, which is a product of lipid peroxidation, using established methods (Kim et al., 2018).

### Long-term lung carcinogenesis study in mice

In the A/J mice diet-based drug regimen study (Figure 4A), four-week-old mice were randomly divided into four groups. Groups 1 and 2 (n = 5 and 13, respectively) were fed a standard diet, while groups 3 and 4 were fed a diet containing metformin and GLSF (n = 10) till the end of the study. Mice in groups 2, 3, and 4 NNK were given two i.p injections of 100 mg/kg of NNK on Days 1 and 8 (Day 1 is the first day of NNK injection). The dose for GLSF obtained from diet consumption was estimated to be 2.0 g/kg, the same as the toxicity study. Metformin was given 250 mg/kg based on a published study (Memmott et al., 2010). After the study completed, the mice were anesthetized with isoflurane. Each mouse’s lung was perfused with saline, harvested, weighed, and then fixed in 10% formalin for histological analysis.

### Histology and immunohistochemistry (IHC) analysis

The lung tissues were fixed in 10% neutral formalin for both the acute and chronic studies, and then embedded in paraffin. The paraffin-embedded tissues were de-paraffinized using xylene and ethanol, and then stained with hematoxylin and eosin (H&E) to analyze the histology. For the IHC tissue section, antigen retrieval buffer (Abcam cat no. ab93678) was used to boil the tissues for 20 min. The expression of different proteins in the lung tissue was determined using the Vectastain Elite ABC universal plus kit (PK-8200). The sections were first incubated with bloxall endogenous enzyme blocking solution for 10 min in order to quench endogenous peroxidase activity, and then rinsed three times (5 min each time) with TBST (0.05% Tween-20). Finally, a blocking solution was applied for 20 min. Then sections were incubated with diluted antibodies, anti-COX-2 (1:200, Cell Signaling Technology), anti-NF-kB (p65) (1:400, Cell Signaling Technology), cleaved-caspase-3 (1:400, Cell Signaling Technology), cleaved-PARP (1:100, Cell Signaling Technology) and VEGF-A (1:125, Cell Signaling Technology) overnight at 4°C in a humid chamber. Further processing was performed according to the instructions of the Detection System. Lung tissue sections were evaluated at 20 and 40x magnification using a Leica DM750 LED Biological Microscope.

### Statistical analysis

The data is presented as either mean ± standard deviation (SD) or mean ± standard error of the mean (SEM). The figure legends explain the data presentation and errors quantification. GraphPad Prism version 9 (La Jolla, CA, United States) was used to create graphs and analyze the data with ANOVA. Statistical significance was determined by a *p*-value of less than 0.05, and the figure legends indicate how the statistical differences are denoted.

## Results

### Effects of GLSF on BPDE-induced transformation of BEAS-2B cells

**FIGURE 1:**
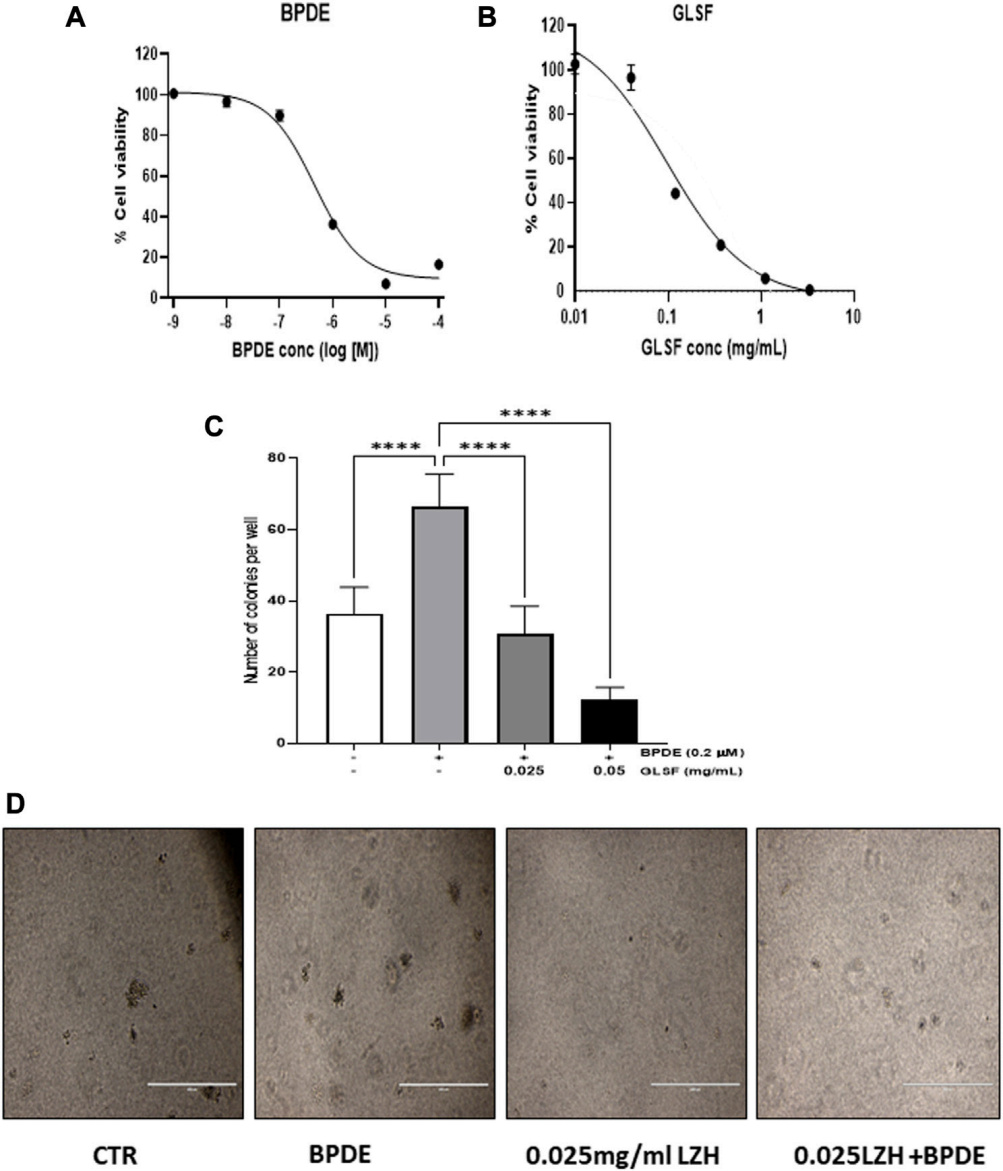
Effects of GLSF gastrointestinal juice extract on BPDE-induced malignant transformation of BEAS-2B cells. **(A,B)** Cell viability of BEAS-2B after being treated with BPDE and GLSF at various doses for 72 h based on SRB cytotoxicity assay. Data plotted are mean ± SD; n = 6. **(C)** The cells were pre-treated with GLSF for 2 h and then treated with 0.2 µM of BPDE for 1 h, and the cells were cultured in the presence of GLSF for 7 days. The cells were then seeded in soft agar in 96-well plate (2000 cells/well) with GLSF in the top layer of agar. Cell colonies were counted under the microscope after 7 days of incubation (n = 6) **(D)** Cell colonies were photographed in each treatment group. ****: *p* < 0.0001.

Since cell transformation is a critical early phase for cancer initiation, investigating the effect of GLSF on cell transformation on BEAS-2B cells which are sensitive to carcinogen-induced transformation *in vitro*, is the step to examining our hypothesis. BEAS-2B cells is a non-tumorous human bronchial epithelial cell line which can be transformed by BPDE (van Agen et al., 1997; Chen et al., 2012; Shahid et al., 2023). In BEAS-2B cells, BPDE induced dose-dependent cytotoxicity, with IC_50_ as 0.46 μM (Figure 1A). GLSF also demonstrated dose-dependent cytotoxicity in BEAS-2B cells, with the IC_50_ value of 0.081 mg/mL (Figure 1B). We further examined the effects of GLSF at a non-toxic concentration on BPDE-induced bronchial epithelial cell transformation. BPDE at 0.2 μM significantly induced colony formation (Figures 1C,D). The gastrointestinal juice extract of GLSF at 0.025 and 0.05 mg/mL alone did not induce colony formation. However, GLSF significantly attenuated BPDE-induced malignant transformation dose-dependently (Figures 1C,D).

### Effects of GLSF on short-term B[a]P-induced lung toxicity in mice

**FIGURE 2:**
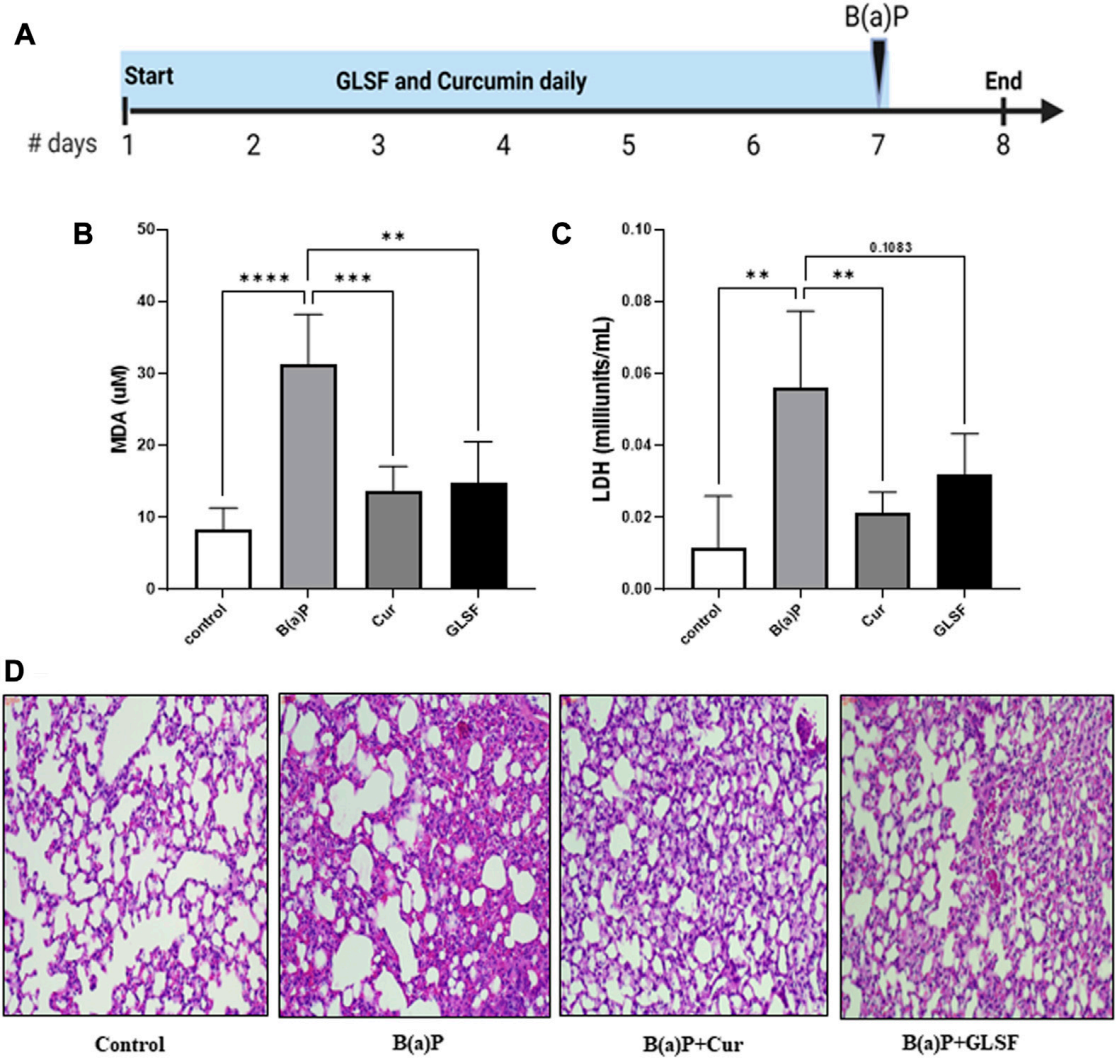
Effect of GLSF treatment on B[a]P-Induced lung toxicity. **(A)** Graphic representation of treatment regimen. **(B)** Effects of GLSF and curcumin on B[a]P-induced plasma levels of malondialdehyde (MDA), a marker to assess lipid peroxidation (LPO). **(C)** Effects of GLSF and curcumin on B[a]P-induced plasma levels of lactate dehydrogenase (LDH), a marker for tissue damage. **(D)** Effects of GLSF on B[a]P-induced histopathologic changes, the H&E staining of mouse lung sections (×20). Data are plotted as mean ± SD. An ordinary one-way ANOVA followed by a Dunnett’s multiple comparison test was used to assess statistical differences. **: *p* < 0.01, ***, *p* < 0.001, ****: *p* < 0.0001.

To determine the effects of GLSF on B[a]P-induced lung toxicity *in vivo*, a single dose of B[a]P was used to induce acute lung toxicity in mice. The experimental design is outlined in Figure 2A. Mice were treated with either GLSF (2 g/kg) or curcumin (100 mg/kg) daily for 7 days and then challenged with a single dose of B[a]P. Malondialdehyde (MDA) content is an important indicator of lung toxicity induced by B[a]P, and lactate dehydrogenase (LDH) activity also is assessed against B[a]P to demonstrate the amount of oxidative damage in mice. A significant increase in the MDA level was observed in B[a]P-treated mice compared to the control group (Figure 2B). Pretreatment of GLSF (2.0 g/kg body weight) significantly decreased lung toxicity at the same level of efficacy as curcumin. We also observed that LDH activity was increased significantly in only B[a]P-treated mice (Figure 2C), pretreatment of GLSF decreased the LDH activity but not significantly, maybe due to a high variation within the group (Figure 2C).

Analysis of lung histology on H&E-stained sections is one of the critical parameters for the analysis of acute toxicity of B[a]P in lungs. The administration of B[a]P to the mice caused significant disruption of the overall lung architecture compared to the control group (Figure 2D). It also caused severe destruction of alveolar architecture, necrosis of the alveolar epithelium, and infiltration of inflammatory cells. GLSF at the dose (2 g/kg) protected against B[a]P-induced lung histological changes. Curcumin at a dose of 100mg/kg also showed a similar degree of protection against B[a]P in terms of lung histology.

### Effects of GLSF on NNK-Induced lung carcinogenesis in mice

**FIGURE 3:**
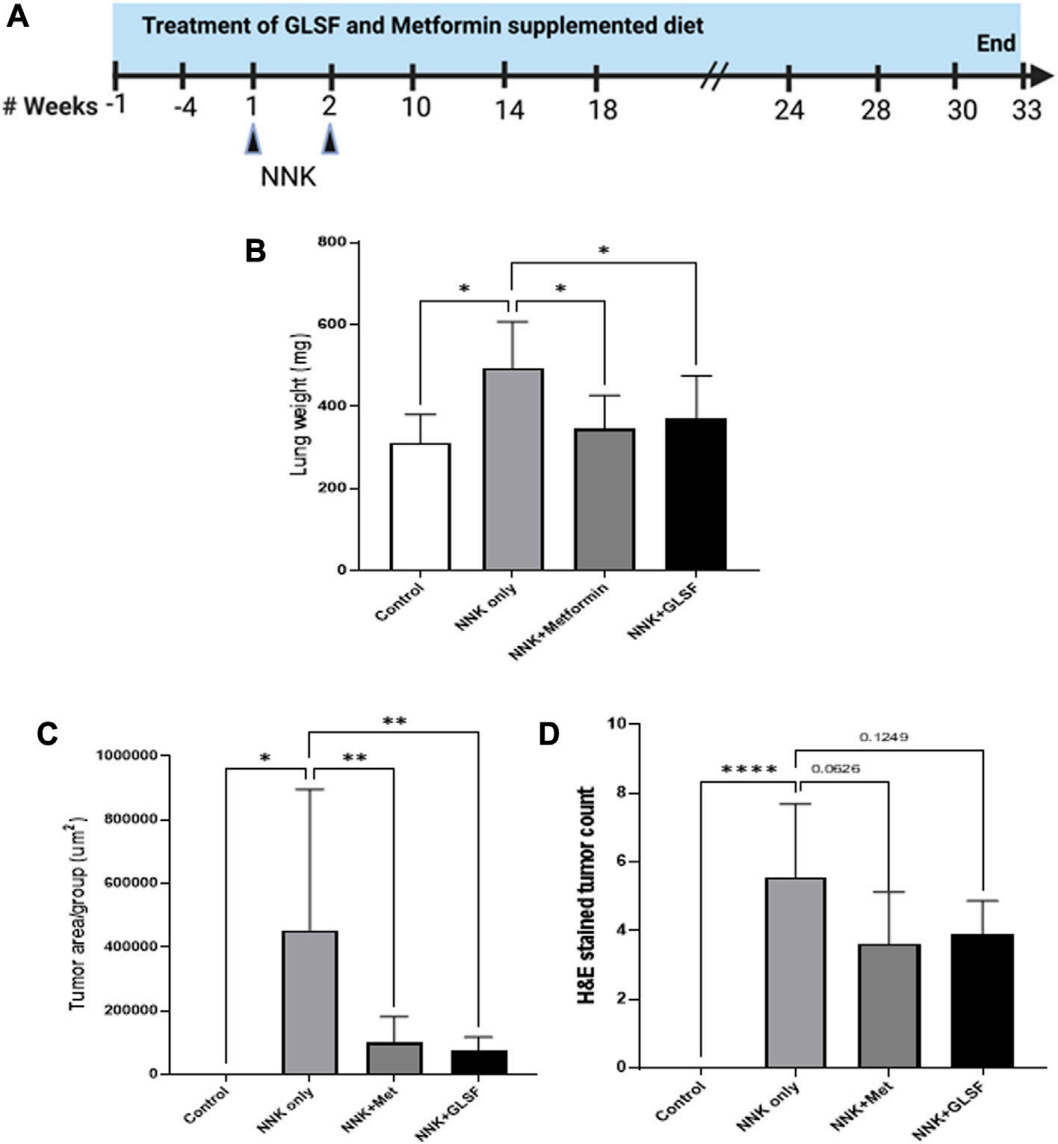
Effects of GLSF on NNK-induced lung carcinogenesis. **(A)**. Experimental design. The A/J mice were divided into four groups: (1) negative control group without NNK exposure (n = 5); (2) NNK only group (n = 13); (3) NNK and treated with metformin (n = 11); (4) NNK and treated with GLSF (n = 10); The drug treatment started 4 weeks before the NNK exposure. **(B)**. Lung weight changes in all the group at the end of study. **(C)** Tumor area per group on the H&E slides. **(D)** Count of tumor numbers on the on the H&E slides. Data are expressed as mean ± SD. An ordinary one-way ANOVA followed by a Dunnett’s multiple comparison test was used to assess statistical differences. *: *p* < 0.05, **: *p* < 0.01, ****p < 0.0001, ns = non-significant.

The NNK is a potent carcinogen that successfully induces lung cancer in A/J mice (Johnson et al., 2008; Aizawa et al., 2013). The lung carcinogenesis model in A/J mice was developed by intraperitoneal injection with two weekly NNK (100 mg/kg) doses, and the experimental design is shown in Figure 3A. Metformin at 250 mg/kg was used as a positive control. Lung weight is an important factor in evaluating tumors and assessing the extent of tumor growth within the lungs. In Figure 3B, NNK-treated mice showed a 58.24% lung weight increment compared to the control. However, treatment with metformin and GFSF decreased lung weight significantly by 29.66% and 24.74%, respectively, suggesting a chemoprotective effect. The tumor area was determined in the H&E-stained lung under a microscope. As expected, NNK significantly induced the lung tumor area (Figure 3C), and GLSF significantly attenuated to a similar degree as metformin (Figure 3C). The number of tumors in H&E-stained slides decreased by 34.77% with metformin and 30.26% with GLSF, compared to only NNK as shown in Figure 3D. However, there was no statistically significant difference which may be attributed to the high variation within the group of mice, as depicted in Figure 3D.

**FIGURE 4:**
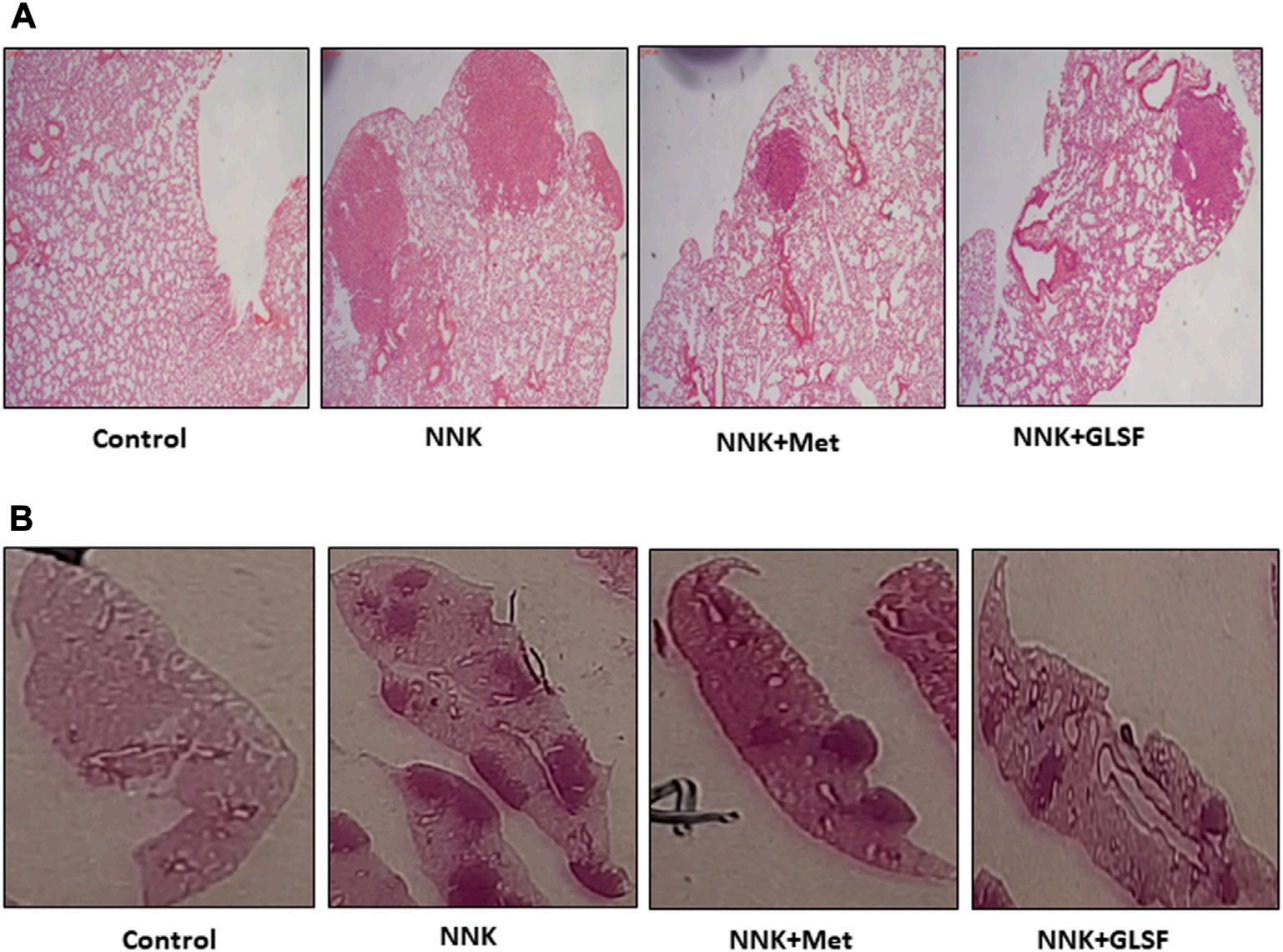
Effects of GLSF on NNK-induced lung carcinogenesis. **(A,B)** Representative H&E-stained cross-sections of lung tissues from four groups, negative control group without NNK exposure; NNK only group; NNK treated with metformin; and NNK treated with GLSF.

**FIGURE 5:**
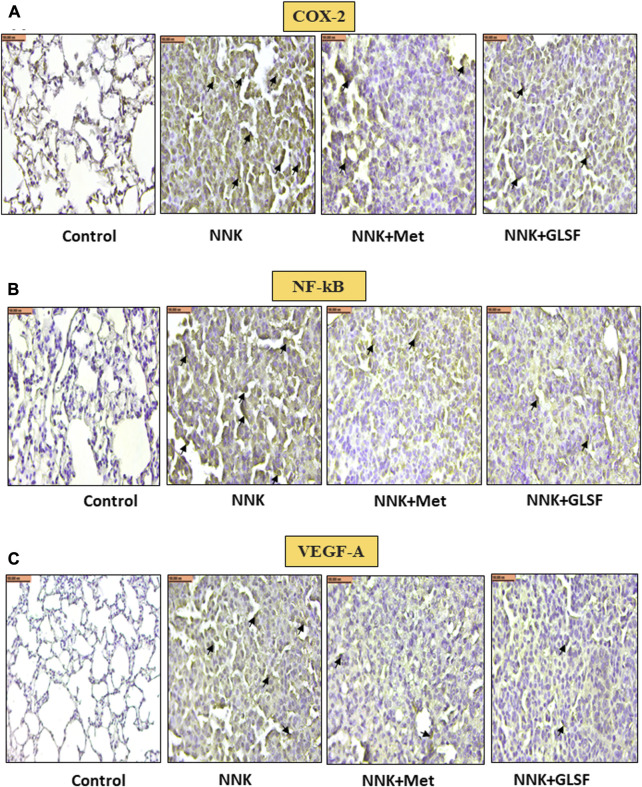
Effects of GLSF on NNK-induced Cox-2, NF-kB, and VEGF-A expression in lung tumors. Representative cross-section images of the lung after immunohistochemical staining with Cox-2 **(A)**, NF-kB **(B)**, and VEGF-A **(C)**. IHC images can provide insights into the effects of GLSF on NNK-induced Cox-2, NF-kB, and VEGF-A expression in lung tumors. In NNK-induced lung tumors, Cox-2, NF-kB, and VEGF-A expression typically increase due to the inflammatory and angiogenesis response. Treatment with GLSF reduces expression, decreasing the intensity or extent of Cox-2, NF-kB, and VEGF-A staining in IHC images. (×40). Scale bar 100 nm (The black arrow shows the cells with expression).

**FIGURE 6:**
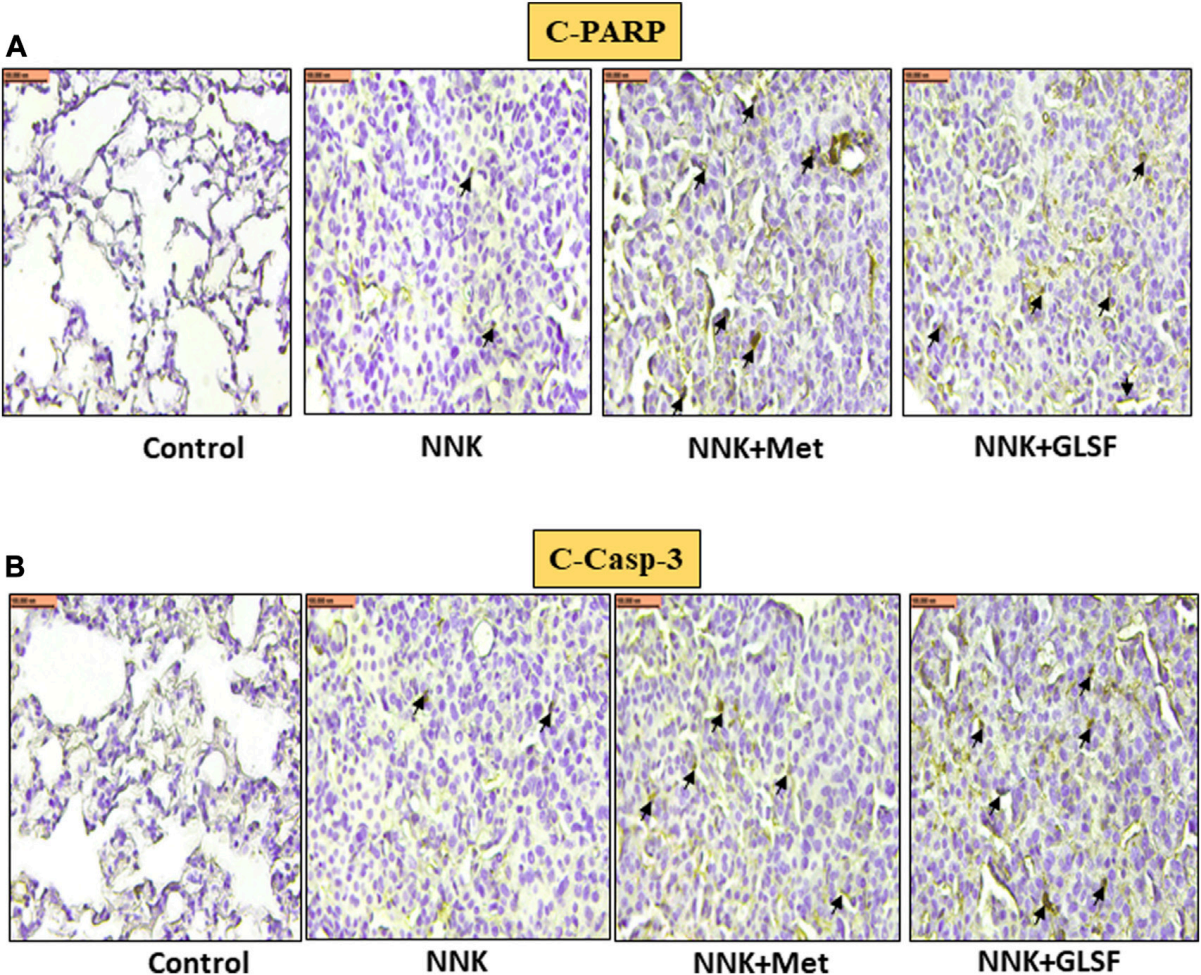
Effects of GLSF on NNK-induced cleaved-PARP and cleaved-caspase-3 expression in lung tumors. Representative cross-section images of the lung after immunohistochemical staining with c-PARP **(A)** and c-caspase-3 **(B)**. In NNK-induced lung tumors, c-PARP and c-caspase-3 expression are typically decreased due to the anti-apoptotic response. Treatment with GLSF increased expression, increasing the intensity or extent of c-PARP and c-caspase-3 staining in IHC images. (×40) Scale bar 100 nm (Black arrow is showing the cells with expression).

Figures 4A,B represent the histological examination of the lung tissue section. The animals in the control group illustrate normal architecture with small uniform nuclei. The NNK group reveals loss of architecture, alveolar damage, and increased tumor formations with more extensive areas (Figure 4A). Metformin and GLSF‐treated mice showed slightly reduced alveolar damage and decreased tumor numbers and area (Figures 4A,B). Since inflammatory and angiogenesis markers play important roles in lung tumor formation, we examined the expression level of important inflammatory and angiogenesis markers, such as Cox-2, NF-kB, and VEGF-A by IHC (Figures 5A–C). These proteins contribute to lung tumor formation and progression (Dudhgaonkar et al., 2009; Yoon et al., 2013; Apte et al., 2019). The expression level of Cox-2, NF-kB, and VEGF-A increased in the NNK group compared to the control, as indicated by the arrow. Treatment with metformin and GLSF reduced the expression of these markers (Figures 5A–C). we also checked the apoptotic markers such as cleaved-caspase-3 and cleaved-PARP in the lung tissues. These pro-apoptotic markers which play a significant role in lung tumors by reflecting the activity of apoptotic pathways and providing insights into tumor cell death and survival (Ellisen, 2011; Noble et al., 2013). NNK treatment decreased the expression of cleaved-caspase-3 and cleaved-PARP in lung tumor tissue (Figures 6A,B), while treatment of GLSF and metformin increased the expression (Figures 6A,B). Based on the results, it suggest that exposure to NNK might lead to an increase in inflammation and angiogenesis, while inhibiting apoptosis and potentially promoting tumor cell survival. However, GLSF and metformin have shown promise in enhancing apoptosis and reducing inflammation and angiogenesis in lung tumor cells, which could contribute to the suppression of tumor growth and progression.

## Discussion

To analyze the effect of GL *in vitro*, we first evaluated the potential inhibitory effect of GL extract (GLSF) on the transformation induced by BPDE (an active form of B[a]P) (Figure 1). The results demonstrated that GLSF exerted an inhibitory effect on transformation in BEAS-2B cells, which suggests that GLSF can potentially mitigate the cellular changes associated with carcinogen-induced transformation in bronchial epithelial cells. The *in vitro* effects of GLSF were verified in short- and long-term mouse models involving B[a]P and NNK, respectively. In the *in vivo* models, it was observed that GLSF not only attenuated B[a]P-induced lung toxicity but also reduced long-term lung cancer development induced by NNK (Figures 2–6). For the first time, the current study demonstrates that GL can directly inhibit the malignant transformation of lung epithelial cells and prevent lung toxicity and carcinogenesis. The findings suggest GL possesses potential anti-cancer properties by targeting key cancer cell growth and survival processes. The findings are consistent with previous studies that provided evidence of the inhibitory effects of GL on cancer development using *in vitro* models (Suarez-Arroyo et al., 2013; Jin et al., 2020). The treatment of GL has shown the ability to inhibit cell division and induce cell death in various cancer cell lines (Cheng et al., 2020).

The B[a]P and NNK treatment resulted in lung toxicity in 24 h and lung cancer in 33 weeks, respectively, as evidenced by toxicity markers assays, histopathological alterations, and lung cancer markers. These alterations are consistent with previously published reports (Sehgal et al., 2013; Breau et al., 2019). In the short-term treatment of any toxicant, including B[a]P, oxidative stress can be evaluated by analyzing the toxicity markers such as LPO and LDH (Liu et al., 2015; Bukowska and Duchnowicz, 2022). LPO is a process mainly caused by oxidative stress, and many studies showed that a notable boost in the production of malondialdehyde (MDA), a lipid peroxidation product, was observed after B[a]P treatment in mice (Liu et al., 2015). Another toxicity marker, LDH, is a serum toxicity marker that discharges extracellularly as a biochemical parameter of cell platelet activation and membrane damage (Jurisic et al., 2015). Our results showed increased MDA and LDH activity levels in B[a]P treated mice. However, pretreatment with GLSF reversed the level of MDA and the activity of LDH (Figure 2). Previous studies also showed that GL could protect the kidneys from damage by maintaining antioxidant molecules decreased by cisplatin toxicity (Mahran and Hassan, 2020). Overall, it suggests that GLSF can potentially mitigate oxidative stress and protect against B[a]P-induced tissue damage. The histological findings supported the above results and showed that the GLSF protects against B[a]P-induced lung toxicity (Figure 2). In this study, curcumin showed the same degree of protective effects as GLSF, which is known to show protective effects against lung inflammation (Sehgal et al., 2012).

Since cancer is a multistep process disease, various factors, including inflammatory, apoptotic, and angiogenic markers, play crucial roles in the initiation and progression of cancer (Aguilar-Cazares et al., 2019; Carneiro and El-Deiry, 2020). In the long-term study, we analyzed several cancer markers to evaluate the chemopreventive effects of GL against NNK-induced lung cancer. Previous studies have provided evidence that GL has the ability to attenuate carcinogenesis by regulating key markers associated with inflammation, apoptosis, and angiogenesis (Stanley et al., 2005; Thyagarajan et al., 2006; Cheng et al., 2020). We found that GLSF treatment decreased the expression of COX-2 and NF-kB significantly compared to the only UV group (Figure 5). Our results indicate that inflammation is a significant target for GLSF; previous studies validate our data (Dudhgaonkar et al., 2009; Yoon et al., 2013). VEGF-A is a cytokine that regulates the formation of new blood vessels from pre-existing vascular networks. Therefore in cancer, it plays an essential role in angiogenesis (Apte et al., 2019). By inhibiting the expression of VEGF-A, GLSF may interfere with the formation of new blood vessels within tumors, ultimately impeding their growth and metastasis.

We made a finding regarding the effects of GLSF treatment on lung tumors induced by NNK in mice. Specifically, we found that GLSF treatment increased the immunostaining of cleaved caspase-3 and cleaved PARP in NNK-induced tumor cells compared to the control mice (Figure 6). This suggests that GLSF stimulates the activation of apoptosis markers in lung tumor cells. Caspase-3 is a critical enzyme involved in the process of apoptosis, which is responsible for programmed cell death and plays a crucial role in eliminating cancer cells (Zhou et al., 2018). Another marker is PARP, which plays a role in DNA repair and cell survival. Elevated levels of PARP immunostaining indicate increased DNA damage response and potentially enhanced cancer cell death (Ellisen, 2011). At the same time, treatment with GLSF and metformin attenuated the cleaved caspase-3 and cleaved PARP expression.

## Conclusion

In conclusion, our data strongly provide evidence that dietary supplements of GLSF may have preventive effects against tobacco smoke carcinogens-induced lung toxicity and tumor development, which may be partly attributed to GLSF’s activity on the modulation of inflammatory and apoptotic pathways. Since GLSF is a natural compound used for decades, the data from the present study indicate that a retrospective clinical trial should be designed to examine if smokers using the GLSF develop fewer lung cancer cases than smokers that do not take GLSF. More cancer cell lines and lower GLSF concentration should be tested before proposing a clinical trial.

## Data Availability

The original contributions presented in the study are included in the article/Supplementary Material, further inquiries can be directed to the corresponding authors.
